# Correction: Registered report: Survey on attitudes and experiences regarding preregistration in psychological research

**DOI:** 10.1371/journal.pone.0315108

**Published:** 2024-12-02

**Authors:** The PLOS ONE Staff

There are errors in the Author Contributions. The correct contributions are:

**Conceptualization:** Lisa Spitzer, Stefanie Mueller

**Data curation:** Lisa Spitzer

**Formal analysis:** Lisa Spitzer

**Investigation:** Lisa Spitzer

**Methodology:** Lisa Spitzer, Stefanie Mueller

**Project administration:** Stefanie Mueller

**Resources:** Stefanie Mueller

**Software:** Lisa Spitzer

**Supervision:** Stefanie Mueller

**Visualization:** Lisa Spitzer

**Writing–original draft:** Lisa Spitzer

**Writing–review & editing:** Stefanie Mueller.

The publisher apologizes for the error.
