# Supplementary material for: Registered report: Survey on attitudes and experiences regarding preregistration in psychological research
Source: PLoS One. 2023 Mar 16;18(3):e0281086. doi: 10.1371/journal.pone.0281086 (PMC10019715; doi:10.1371/journal.pone.0281086)
Supplement: S1 File — The questionnaire is displayed in a PDF for easy inspection. Please note that filters do not work in this view, i.e., all items are displayed. (PDF) [file pone.0281086.s004.pdf]

Page 01

Welcome

Dear reader,

This survey is part of the meta-scientific research that is currently underway at the **Leibniz Institute for Psychology (ZPID)**. We are an open science institute located in Germany, and we strive to support researchers in psychology and related fields by providing an excellent (digital) infrastructure adapted to their needs.

With this survey, we hope to learn more about **preregistration in psychology**. Preregistration of research is becoming more prominent in psychology as it has been suggested as a means to reduce publication bias and questionable research practices. We are interested in your motivation for (as well as your reasons against) preregistering your research and the obstacles and disadvantages you may have encountered or expect to encounter.

We would greatly appreciate learning about your views on preregistration (both if you have or have not actively preregistered your work before). The survey takes about 20-25 minutes to complete. All your responses will be treated confidentially, and anonymity will be preserved. At the end of the survey, you will be given the opportunity to participate in a drawing to win one of 40 gift cards for Amazon or Barnes & Noble worth 50€ (approx. $56).

Participant Information and Informed Consent

Overall information: We invite you to participate in our survey of preregistration in psychology. Your participation in this survey is voluntary. You may choose not to participate. The purpose of this form is to provide you with all information necessary to decide if you want to participate. Please read it carefully. If you have any questions that are not answered by this form, please do not hesitate to contact us (see below).

Aim of this survey: This survey was designed to investigate the current attitudes of psychology researchers toward preregistration. We want to gain more knowledge about this topic in order to improve our aids and services for preregistration.

Procedure: In the following, you will see statements that inquire about your different attitudes toward and experiences with preregistration. Response formats for the items include rating scales, single or multiple choice, or open text input. The survey will take about 20-25 minutes to complete. Participants that do not belong to the target sample will be excluded at the beginning of the survey.

Collection and processing of personal information: We collect data on age, gender, country, degree, and research topic. Based on these data, it will not be possible to identify specific persons. Your email address (optional for receiving further information or participating in the drawing) is collected separately, i.e., it is not possible for us to link it in any way to your responses in the survey. Furthermore, we do not collect other identifying information such as your name or IP address. Anonymity is therefore maintained.

Possible risks: No known risks are associated with participation in this survey.

Withdraw participation: Participation is voluntary. You have the right to refuse participation and withdraw at any time without any consequence whatsoever and without stating any reasons for doing so. Yet participation in the drawing is only possible if the survey has been completed.

Data processing and usage of anonymized data: Anonymity of your responses is preserved, and no individual association will be possible, as email addresses will be collected separately. Your data will be processed in agreement with the legal provisions of the European law (General Data Protection Regulation, more information is available here: https://gdpr.eu/tag/gdpr/). The results of our survey will presumably be published as a scientific research article. Furthermore, the anonymized data will be made publicly available in line with transparent and open research practices, as recommended by the German Research Foundation (DFG) and the German Psychological Society (DGPs).

Contact address: Lisa Spitzer, PhD Student at the Leibniz Institute for Psychology (ZPID), ls@leibniz-psychology.org, +49 (0) 651 201-2603

Informed consent: I have received the participant information about the aims and procedure of the survey, read it carefully, and fully understood it. If I have any more questions, I know that I can contact the examiner. I have had enough time to decide for or against participation in this survey. By selecting “I agree with these conditions” below, I declare that I understood this information and want to participate voluntarily in this survey. I have understood that I can withdraw my participation in the survey at any point without stating any reasons for doing so, and without any negative consequences.

○ I agree with these conditions.

Please answer the following question to verify that you are not a bot.

six plus five is ______

What is your gender?

[Please choose]

How old are you?

years

Which country do you live in?

All country names are displayed in English.

[Please choose]

How many years have you been working in (psychological) research?

years

What is your highest degree (in psychology)?

- ◯ Bachelor's degree
- ◯ Master's degree
- ◯ Doctoral degree
- ◯ Habilitation and/or full professorship
- ◯ Other:

Do you plan to continue your academic career, or leave academia?

If you are unsure, please indicate your current tendency here.

- ◯ I plan to continue my academic career.
- ◯ I consider leaving academia.
- ◯ Other:

Does your research or studies fall within the scope of psychology?

- ◯ yes
- ◯ no

What is your main research topic (or what is your main interest in psychology)?

Please select every option that applies to you.

- ☐ Organizational psychology
- ☐ Social psychology
- ☐ Educational psychology
- ☐ Developmental psychology
- ☐ General psychology
- ☐ Neuroscience/neuropsychology
- ☐ Clinical psychology
- ☐ Differential psychology
- ☐ Experimental/cognitive psychology
- ☐ Research methods
- ☐ Other: ____________
- ☐ Psychology is not my main field.

Page 04

Quota

Page 05

Quota stop

Thank you for your willingness to participate in our survey!

In this survey, we want to examine psychological researchers' current experiences and atittudes regarding preregistrations. For this purpose, we want to inquire attitudes by psychological researchers with either a bachelor's degree, master's degree, doctoral degree, or habilitation / full professorship. We have defined specific quotas to represent these groups appropriately. As we already reached the maximum number of participants for your academic group, you cannot participate further.

Nevertheless, we are very thankful for your contribution!

Page 06

Def

To ensure a common understanding among the participants, we define preregistration as follows.

A preregistration is a study plan that is registered with an independent party (e.g., a repository) before the data has been collected or examined. Upon registration, a time-stamped record of the study plan is created that will eventually be published (possibly after an embargo period). If the study plan changes afterwards, either a new record needs to be created or the deviations will be apparent when comparing the preregistration to the final manuscript. Thus, preregistration allows a transparent presentation of what was planned at a certain time point and what changes may have been made to a study till its publication. (e.g., Nosek et al., 2018).

Please bear this definition in mind when answering the questions.

Which aspects belong to the definition of “preregistration”, based on the definition on the previous page?

This question’s purpose is to check if you understood our definition of “preregistration” correctly. There are, of course, many ways to define preregistration. However, please base your answer on our definition and keep this definition in mind when answering the survey.
Please select every correct option.

- ☐ Preregistrations are assigned a time stamp.
- ☐ Preregistration aims at increasing the transparency of potential changes made to a study.
- ☐ Preregistration is typically done after data analysis.
- ☐ A study must be conducted exactly as it was preregistered.
- ☐ Preregistrations are publicly accessible (possibly after an embargo period).

text('G020')

Unfortunately, you did not answer the control question correctly.

These aspects do belong to the definition of preregistration:

- Preregistrations are assigned a time stamp.
- Preregistrations are publicly accessible (possibly after an embargo period).
- Preregistration increases the transparency of potential changes made to a study.

These aspects do not belong to the definition of preregistration:

- A study must be conducted exactly as it was preregistered. *The preregistration document itself cannot be changed, yet changes to the study are possible which should then be disclosed in an additional deviations file or in the final manuscript.*
- Preregistration is typically done after data analysis. *Preregistration is done before data collection (or before data analysis if it can be shown that authors did not have yet access to the data).*

Please review our definition:

A preregistration is a study plan that is registered with an independent party (e.g., a repository) before the data has been collected or examined. Upon registration, a time-stamped record of the study plan is created that will eventually be published (possibly after an embargo period). If the study plan changes afterwards, either a new record needs to be created or the deviations will be apparent when comparing the preregistration to the final manuscript. Thus, preregistration allows a transparent presentation of what was planned at a certain time point and what changes may have been made to a study till its publication. (e.g., Nosek et al., 2018).

Please bear this definition in mind when answering the questions.

Have you preregistered a study before?

- ◯ yes
- ◯ no

How many studies have you preregistered before?

[ ]

Where did you learn about preregistration?

Please select every option that applies to you.

- ☐ Official event at workplace (e.g., colloquium, talk)
- ☐ Lecture at university
- ☐ Informal conversation with colleagues/peers
- ☐ Supervisor
- ☐ Project at university
- ☐ I don’t know.
- ☐ I have not heard about preregistration before this survey.
- ☐ Other: [ ]

What is your preferred preregistration template (i.e., form that lists important elements to preregister and can be used by researchers to create their own preregistration)?

- ◯ Replication Recipe (Brandt et al., 2013)
- ◯ Center for Open Science Preregistration Challenge Template (OSF)
- ◯ OSF-Standard Pre-Data Collection Registration
- ◯ AsPredicted
- ◯ van ‘t Veer & Giner-Sorolla (2016)
- ◯ Psychological Research Preregistration-Quantitative (PRP-QUANT) Template
- ◯ I don’t use templates.
- ◯ I don’t know.
- ◯ Other template: [ ]

Why do you prefer the selected template?

Please select every option that applies to you.

- ☐ It is the only template I know
- ☐ It is comprehensive
- ☐ It is easy to use
- ☐ It is preferred by my co-authors/colleagues/peers
- ☐ It is time-efficient
- ☐ It was the first template I used
- ☐ It fits well with my research area
- ☐ It is recommended by my supervisor
- ☐ Other: ______

When I preregister, I use the following repository (i.e., uploading platform) for sharing my preregistration:

Please select every option that applies to you.

- ☐ OSF
- ☐ Personal website
- ☐ Institutional website
- ☐ Offline (e.g., I only share it with my co-authors or store it at my institution)
- ☐ PsychArchives (ZPID)
- ☐ AsPredicted
- ☐ Other: ______

Which persons/institutions influence your decision for or against preregistration?

Please select every option that applies to you.

- ☐ Editors
- ☐ Funding guidelines
- ☐ Peers/colleagues
- ☐ Institute guidelines
- ☐ Supervisor
- ☐ Co-authors
- ☐ None
- ☐ Other: ______

When you read a paper describing a study that was preregistered – do you read the corresponding preregistration?

- ◯ Yes, always
- ◯ Most of the time
- ◯ Rarely
- ◯ Never
- ◯ I can’t recall reading a paper that was preregistered.

What do you think will be the long-term *positive* consequences if researchers are obliged to publicly preregister their studies?

What do you think will be the long-term *negative* consequences if researchers are obliged to publicly preregister their studies?

Please indicate your opinion regarding the below-listed items.

	Strongly disagree	Disagree	Slightly disagree	Neither agree nor disagree	Slightly agree	Agree	Strongly agree
I think that preregistration does **not** improve research significantly.	○	○	○	○	○	○	○
Preregistration cannot prevent questionable research practices.	○	○	○	○	○	○	○
I want to be part of the open science movement.	○	○	○	○	○	○	○
It would be easy for me to preregister my studies.	○	○	○	○	○	○	○
I have more trust in researchers who preregister their studies than in those who don't.	○	○	○	○	○	○	○
Preregistration of studies should be obligatory.	○	○	○	○	○	○	○
My co-authors and supervisors want me to preregister my studies.	○	○	○	○	○	○	○
I have more trust in research findings when the study has been preregistered.	○	○	○	○	○	○	○
Science profits from preregistration.	○	○	○	○	○	○	○
My peers and colleagues motivate me to preregister my studies.	○	○	○	○	○	○	○
In selection decisions people who preregister their studies should be preferred (given the same qualification).	○	○	○	○	○	○	○
I know how to create and upload a preregistration.	○	○	○	○	○	○	○
Some researchers exploit preregistration (e.g., by cheating).	○	○	○	○	○	○	○
I will preregister my studies in the near future.	○	○	○	○	○	○	○

Do you have any comments regarding the listed items?

For example, do you want to indicate your opinion in more detail, or do you have any other remarks?

Please indicate your opinion regarding the below-listed items.

	Strongly disagree	Disagree	Slightly disagree	Neither agree nor disagree	Slightly agree	Agree	Strongly agree
Preregistration can prevent publication bias (i.e., only publishing positive/significant results).	○	○	○	○	○	○	○
With the current implementation of preregistration, it is my decision if I want to preregister my studies.	○	○	○	○	○	○	○
Preregistration is important for me.	○	○	○	○	○	○	○
Preregistration improves a study’s quality.	○	○	○	○	○	○	○
The costs of preregistering a study are higher than the usefulness of preregistering it.	○	○	○	○	○	○	○
Even if I wanted to preregister, there are external factors that hinder me (e.g., my co-authors or supervisors do not want to preregister).	○	○	○	○	○	○	○
I intend to preregister my studies in the future.	○	○	○	○	○	○	○
Preregistration can prevent selective reporting (i.e., only reporting variables that yielded significant results).	○	○	○	○	○	○	○
In today’s psychological research community, it is deemed necessary to preregister.	○	○	○	○	○	○	○
Preregistration can prevent *p*-hacking (i.e., misusing data analyses to find patterns that can be presented as statistically significant).	○	○	○	○	○	○	○
Preregistering studies is generally unnecessary.	○	○	○	○	○	○	○
Preregistration is highly acknowledged in psychological science.	○	○	○	○	○	○	○
I feel social pressure to preregister my studies.	○	○	○	○	○	○	○
Preregistration increases the credibility of psychological research.	○	○	○	○	○	○	○
Preregistration is a bad initiative.	○	○	○	○	○	○	○

Do you have any comments regarding the listed items?

For example, do you want to indicate your opinion in more detail, or do you have any other remarks?

Please indicate your opinion regarding the below-listed items.

	Strongly disagree	Disagree	Slightly disagree	Neither agree nor disagree	Slightly agree	Agree	Strongly agree
Preregistration makes science more transparent.	○	○	○	○	○	○	○
Preregistration is **not** useful in practice.	○	○	○	○	○	○	○
Preregistration hinders exploratory research.	○	○	○	○	○	○	○
I want to use preregistration in the future.	○	○	○	○	○	○	○
I think that many researchers preregister their studies.	○	○	○	○	○	○	○
I feel that nowadays, a great variety of new structures help creating preregistrations, for example templates or repositories (i.e., uploading platforms).	○	○	○	○	○	○	○
I don’t feel well informed about preregistration.	○	○	○	○	○	○	○
Preregistration is very useful.	○	○	○	○	○	○	○
A preregistration badge (i.e., a public acknowledgment that a study was preregistered provided by many journals) increases my trust in a study.	○	○	○	○	○	○	○
I feel social pressure **not** to preregister my studies.	○	○	○	○	○	○	○
Science should be open and transparent.	○	○	○	○	○	○	○
Preregistration decreases scientific progress.	○	○	○	○	○	○	○

Do you have any comments regarding the listed items?

For example, do you want to indicate your opinion in more detail, or do you have any other remarks?

Please indicate your opinion regarding the below-listed items.

	Strongly disagree	Disagree	Slightly disagree	Neither agree nor disagree	Slightly agree	Agree	Strongly agree
Given the present scientific context, preregistering my studies hinders my career.	○	○	○	○	○	○	○
Preregistration helps me to plan my study in more detail.	○	○	○	○	○	○	○
I have the feeling that nowadays, it is harder to publish studies that were not preregistered.	○	○	○	○	○	○	○
I feel morally obligated to preregister my studies.	○	○	○	○	○	○	○
Preregistration causes considerable time cost.	○	○	○	○	○	○	○
I don’t like that preregistration limits my flexibility.	○	○	○	○	○	○	○
I am afraid that after preregistering my studies others will find errors or deviations in/from my study plans.	○	○	○	○	○	○	○
I feel like preregistration is an investment in my future (e.g., it is helpful for my career).	○	○	○	○	○	○	○
I am afraid that I could have a competitive disadvantage when I preregister my studies.	○	○	○	○	○	○	○
The preregistration badge (i.e., a public acknowledgment that a study was preregistered provided by many journals) would be an incentive for me to preregister my studies.	○	○	○	○	○	○	○

Do you have any comments regarding the listed items?

For example, do you want to indicate your opinion in more detail, or do you have any other remarks?

Please indicate your opinion regarding the below-listed items.

	Strongly disagree	Disagree	Slightly disagree	Neither agree nor disagree	Slightly agree	Agree	Strongly agree
I use preregistration because it represents good scientific practice for me.	◯	◯	◯	◯	◯	◯	◯
I would be afraid of scooping (i.e., someone taking my idea and publishing it before me) when preregistering my study.	◯	◯	◯	◯	◯	◯	◯
For me, there are not enough incentives to preregister my studies.	◯	◯	◯	◯	◯	◯	◯
Preregistration helps making my studies more transparent.	◯	◯	◯	◯	◯	◯	◯
For my projects, preregistration is unnecessary.	◯	◯	◯	◯	◯	◯	◯
Preregistration slows down the scientific progress of my project.	◯	◯	◯	◯	◯	◯	◯
I want others to be able to comment on my planned studies.	◯	◯	◯	◯	◯	◯	◯
I think planning studies in detail in the context of preregistration is fun.	◯	◯	◯	◯	◯	◯	◯
I am unsure about confidentiality issues and intellectual property rights when preregistering my studies.	◯	◯	◯	◯	◯	◯	◯
It is necessary that preregistration limits my flexibility.	◯	◯	◯	◯	◯	◯	◯

Do you have any comments regarding the listed items?

For example, do you want to indicate your opinion in more detail, or do you have any other remarks?

Please indicate your opinion in the below-listed questions.

Answering these questions is optional, but would be greatly appreciated.

What caused you to do your first preregistration?
Please select every option that applies to you.

- ☐ Requirement to get funding
- ☐ Suggestion by my supervisor
- ☐ Recommendation of co-authors for a specific project
- ☐ Preregistration was mandatory for a project
- ☐ Informal conversation with colleagues/peers
- ☐ Self-motivated
- ☐ Other (please indicate all other situations/persons that motivated you to do your first preregistration):

Did your motivation to preregister change over time? If yes, how?

- ○ I am now **more** motivated to preregister than I was before because:
- ○ I am now **less** motivated to preregister than I was before because:
- ○ My motivation did not change over time.

What do you perceive as benefits of preregistrations?

What do you perceive as drawbacks of preregistration?

Did you encounter specific problems when preregistering a study? If yes, which ones?

Please select every option that applies to you.

- [ ] I found it problematic to not have flexibility during my analyses
- [ ] Deviations were necessary and my study lost credibility
- [ ] Conflict with supervisor/co-author
- [ ] I got scooped (i.e., someone took my idea and published it before me)
- [ ] I was insecure about what needs to be included in the preregistration
- [ ] It took very long to do the preregistration
- [ ] Study design would have needed to be changed because details did not work, but this was not possible
- [ ] Errors in the preregistration could not be changed afterwards
- [ ] None
- [ ] Other (please indicate all other problems you encountered):

What worries do you have with respect to preregistering your studies?

Please select every option that applies to you.

- [ ] I would be insecure about what needs to be included in the preregistration
- [ ] My supervisor/co-author(s) would object
- [ ] If deviations were necessary, my study would loose credibility
- [ ] Errors in the preregistration cannot be changed afterwards
- [ ] Scooping (i.e., someone taking my idea and publishing it before me)
- [ ] High time costs
- [ ] Low flexibility
- [ ] Maybe the study design would need to be changed because details do not work, but this would not be possible
- [ ] None
- [ ] Other (please indicate all other worries you have):

What are reasons for you not to preregister your studies?

Please indicate your opinion in the below-listed questions.

Answering these questions is optional, but would be greatly appreciated.

Regarding preregistration templates, what do you prefer?

- ◯ A more restricted template that gives a lot of suggestions and reminds you of left-out information
- ◯ A more open preregistration template with open text input, where you are free to write what you want
- ◯ Other: __________
- ◯ I don’t use templates at all, and rather just write my own text.

How would you prefer creating your preregistration?

- ◯ I would prefer a more automated, computer-assisted process (e.g., filling in boxes that are automatically presented).
- ◯ I would prefer a more open, self-administered process (e.g., word document that you can alter, e.g., its structure, to fit your needs).
- ◯ Other: __________

Do you have suggestions how to increase your (and other researchers’) motivation to preregister?

Do you have suggestions how to decrease your (and other researchers’) perceived obstacles regarding preregistration?

What do you think should be improved about preregistration?

Please write down all spontaneous ideas regarding the different possible areas of improvement. Answering is optional, not all fields must be filled out.

Improvements regarding ...

Template:	
Repositories (uploading platforms) and publication:	
Review Process:	
Integration of preregistrations in published articles:	
Education regarding preregistration:	

Page 17

C

Do you have any further comments about this survey?

Planning, conducting, and analyzing a survey is associated with considerable time and financial costs. Therefore, we would like to ask you to submit your data only if you completed the survey faithfully.

Your response will not have any consequences for your participation in the gift card drawing.

- ◯ Yes, my responses can be used in the analyses
- ◯ No, my responses should **not** be used in the analyses

Page 18

L

Drawing & Further Information

Your email address will be saved separately from all other data and no junction will be possible, thus anonymity is preserved. Your email address will be treated with strict confidentiality.

We will delete all email addresses of persons that want to participate in the gift card drawing after the drawing is completed, and all email addresses of persons that want to receive a preprint after the preprint was published and sent, but at the latest after 12 months.

As soon as you select an option, you can enter your email address.

- ☐ I would like to participate in the **drawing to win one of 40 gift cards worth 50€ (approx. $56)**. I agree that my email address will be saved until the winner is drawn. My survey responses will continue to be anonymous and my email address will not be passed on to third parties.
- ☐ I am interested in the results of this study. Please send me a **preprint** by email.

Thank you very much for your participation!

Your responses will help us to gain a more profound insight into the way researchers currently think about preregistration, what experiences they made while preregistering their studies, and what obstacles exist.

Furthermore, we plan to investigate these questions:
1) What influences researchers' intention to use preregistration?
2) Does the research experience influence attitudes and the perceived strength of motivations and obstacles regarding preregistration?

We would like to use the gained knowledge to improve upon the preregistration initiative. Thus, your response is a valuable contribution to the improvement of psychological science!

If you would like to receive further information or have questions, you can send an email to Lisa Spitzer (ls@leibniz-psychology.org).
