# Supplementary material for: Registered report: Survey on attitudes and experiences regarding preregistration in psychological research
Source: PLoS One. 2023 Mar 16;18(3):e0281086. doi: 10.1371/journal.pone.0281086 (PMC10019715; doi:10.1371/journal.pone.0281086)
Supplement: S1 Text — A plain language summary is provided to increase the accessibility of these research findings to a wider audience. It is available in English and German. (DOCX) [file pone.0281086.s005.docx]

Supporting information to ‘Registered Report: Survey on attitudes and experiences regarding preregistration in psychological research’:

**S1: Plain language summary**

Lisa Spitzer^1^ & Stefanie Mueller^1^

^1^ Leibniz Institute for Psychology

**English version**

**Why did we do the study?**

“Preregistration” means that researchers write down everything they have planned for their study. They publish this plan before sampling/analyzing the data when they cannot be influenced by the results yet. This increases the objectiveness. It can also increase the transparency because others can check if the researchers followed their plan, and track deviations from it. However, few psychologists use preregistration. In this study, we explored possible reasons for this. This way, we wanted to find ways to make preregistration more popular.

**What did we do?**

We conducted an online study in which we asked psychologists what they thought about preregistration. We were interested in their attitudes, their motivations to preregister, and what holds them back from doing so. We also asked our participants for suggestions to improve preregistration. Additionally, we examined these two questions: What factors influence the intention to preregister? And how does the amount of time someone has worked in research affect their attitudes toward preregistration?

**What did we find?**

Overall, researchers had positive attitudes toward preregistration. Most of our participants had already preregistered or planned to preregister their studies in the future. The main reasons not to preregister were time and effort.

Generally, our study participants intended to use preregistration more if they

1. had positive attitudes about it
2. felt that others are supportive
3. believed they have the resources and skills to do so
4. believed that preregistration is important.

The shorter someone had been working in research, the more positive their attitudes and motivation were toward preregistration. However, this had no influence on how they perceived obstacles to preregistration.

**What now?**

Our results show that psychological researchers think positively about preregistration. However, we identified some obstacles that should be addressed. For example, our participants found that preregistration is difficult and takes a long time. Better education can help make it easier and faster. Additionally, the work researchers put in their preregistration should be better rewarded. We propose solutions to the identified obstacles. These could help to make preregistration easier and more popular.

**German version / Deutsche Version**

**Warum haben wir die Studie durchgeführt?**

"Prä-Registrierung" bedeutet, dass Forschende alles aufschreiben, was sie für ihre Studie geplant haben. Sie veröffentlichen diesen Plan vor der Sammlung/Analyse der Daten, wenn sie noch nicht von den Ergebnissen beeinflusst sind. Das erhöht die Objektivität. Es kann auch die Transparenz erhöhen, da andere überprüfen können, ob die Forschenden sich an ihren Plan gehalten haben. Außerdem können sie Abweichungen davon nachvollziehen. Allerdings nutzen nur wenige Psycholog*innen Prä-Registrierung. In dieser Studie haben wir mögliche Gründe dafür untersucht. Auf diese Weise wollten wir Wege finden, Prä-Registrierung populärer zu machen.

**Was haben wir gemacht?**

Wir haben eine Online-Studie durchgeführt, bei der wir Psycholog*innen zu ihrer Meinung über Prä-Registrierung befragt haben. Uns interessierten ihre Einstellungen, ihre Motivationen, und was sie davon abhält zu prä-registrieren. Wir fragten unsere Teilnehmer*innen auch nach Vorschlägen zur Verbesserung von Prä-Registrierung. Außerdem untersuchten wir diese beiden Fragen: Welche Faktoren beeinflussen die Absicht, zu prä-registrieren? Und wie beeinflusst die Zeit, die jemand in der Forschung gearbeitet hat, die Einstellung zur Prä-Registrierung?

**Was haben wir herausgefunden?**

Insgesamt waren die psychologischen Forschenden Prä-Registrierung gegenüber positiv eingestellt. Die meisten unserer Teilnehmer*innen hatten schon prä-registriert oder planten, in Zukunft zu prä-registrieren. Die Hauptgründe gegen Prä-Registrierung waren Zeit und Aufwand.

Unsere Teilnehmenden hatten eher die Absicht, Prä-Registrierung zu nutzen, wenn sie

1. eine positive Einstellung dazu hatten
2. das Gefühl hatten, dass andere Prä-Registrierung ebenfalls unterstützen
3. glaubten, dass sie über die nötigen Ressourcen und Fähigkeiten verfügen, um dies zu tun
4. glaubten, dass Prä-Registrierung wichtig ist.

Je kürzer jemand in der Forschung gearbeitet hat, desto positiver waren dessen Einstellung und Motivation gegenüber Prä-Registrierung. Das hatte aber keinen Einfluss darauf, wie Forschende Hindernisse der Prä-Registrierung wahrnahmen.

**Was nun?**

Unsere Ergebnisse zeigen, dass psychologische Forschende Prä-Registrierung im Allgemeinen positiv finden. Trotzdem haben wir einige Hindernisse festgestellt, die gelöst werden sollten. Zum Beispiel fanden unsere Teilnehmenden, dass Prä-Registrierung aufwändig ist und lange dauert. Eine bessere Ausbildung könnte helfen, Prä-Registrierung einfacher und schneller zu machen. Außerdem sollte die Arbeit, die Forschende in die Prä-Registrierung stecken, besser belohnt werden. Wir schlagen verschiedene Lösungen für die festgestellten Hindernisse vor. Diese könnten helfen, Prä-Registrierung einfacher und beliebter zu machen.
