# Supplementary material for: Registered report: Survey on attitudes and experiences regarding preregistration in psychological research
Source: PLoS One. 2023 Mar 16;18(3):e0281086. doi: 10.1371/journal.pone.0281086 (PMC10019715; doi:10.1371/journal.pone.0281086)
Supplement: S2 Text — Power analyses for both hypotheses are presented here. For hypothesis 1, power analyses were performed for the entire model as well as for the individual predictors. (DOCX) [file pone.0281086.s006.docx]

Supporting information to ‘Registered Report: Survey on attitudes and experiences regarding preregistration in psychological research’:

**S6: Power analyses**

Lisa Spitzer^1^ & Stefanie Mueller^1^

^1^ Leibniz Institute for Psychology

# Test for Hypothesis 1: Overall

## Effect sizes from literature

- $R^{2}=0.39$ [1]
- $R^{2}=0.39$ [2]
- $R^{2}=0.443$ [3]
- $R^{2}=0.41$ [4]
- $R^{2}=0.304$ [5] 🡪 chosen for power analysis

## Power analysis

- F tests - Linear multiple regression: Fixed model, *R²* deviation from zero
- Analysis: A priori: Compute required sample size
- Input: Effect size *f²* = 0.4367816
  - *α* err prob = 0.05
  - Power (1 – *β* err prob) = 0.95
  - Number of predictors = 6
- Output: Noncentrality parameter *λ* = 24.0229880
  - Critical *F* = 2.2946013
  - Numerator df = 6
  - Denominator df = 48
  - Total sample size = 55
  - Actual power = 0.9524763

# Test for Hypothesis 1: Attitudes as predictor

## Effect sizes from literature

- $R^{2}=0.24$ [1]
- $R^{2}=0.3364$ [2]
- $\rho^{2}=0.3249$ [3]
- $R^{2}=0.2116$ [4] 🡪 chosen for power analysis

## Power analysis

- t tests - Linear multiple regression: Fixed model, single regression coefficient
- Analysis: A priori: Compute required sample size
- Input: Tail(s) = On
  - Effect size *f²* = 0.2683917
  - *α* err prob = 0.05
  - Power (1 – *β* err prob) = 0.95
  - Number of predictors = 6
- Output: Noncentrality parameter *δ* = 3.3574472
  - Critical *t* = 1.6895725
  - Df = 35
  - Total sample size = 42
  - Actual power = 0.9501825

# Test for Hypothesis 1: Subjective norm as predictor

## Effect sizes from literature

- $R^{2}=0.12$ [1]
- $R^{2}=0.1936$ [2]
- $\rho^{2}=0.16$ [3]
- $R^{2}=0.1156$ [4] 🡪 chosen for power analysis

## Power analysis

- t tests - Linear multiple regression: Fixed model, single regression coefficient
- Analysis: A priori: Compute required sample size
- Input: Tail(s) = One
  - Effect size *f²* = 0.1307101
  - *α* err prob = 0.05
  - Power (1 – *β* err prob) = 0.95
  - Number of predictors = 6
- Output: Noncentrality parameter *δ* = 3.3332204
  - Critical *t* = 1.6646246
  - Df = 78
  - Total sample size = 85
  - Actual power = 0.9514666

# Test for Hypothesis 1: Perceived behavioral control as predictor

## Effect sizes from literature

- $R^{2}=0.18$ [1]
- $R^{2}=0.0441$ [2] 🡪 chosen for power analysis
- $\rho^{2}=0.2916$ [3]
- $R^{2}=0.2116$ [4]

## Power analysis

- t tests - Linear multiple regression: Fixed model, single regression coefficient
- Analysis: A priori: Compute required sample size
- Input: Tail(s) = One
  - Effect size *f²* = 0.0461345
  - *α* err prob = 0.05
  - Power (1 – *β* err prob) = 0.95
  - Number of predictors = 6
- Output: Noncentrality parameter *δ* = 3.2996579
  - Critical *t* = 1.6515348
  - Df = 229
  - Total sample size = 236
  - Actual power = 0.9500185

# Test for Hypothesis 2: Three multiple regression models

## Effect size from literature

- $\eta^{2}=.05$ [6], the corresponding *F* value was used to calculate *R²*

## Power analysis

- t tests - Linear multiple regression: Fixed model, single regression coefficient
- Analysis: A priori: Compute required sample size
- Input: Tail(s) = Two
  - Effect size *f²* = 0.0557895
  - *α* err prob = 0.0167
  - Power (1 – *β* err prob) = 0.95
  - Number of predictors = 2
- Output: Noncentrality parameter *δ* = 4.0637042
  - Critical *t* = 2.4070603
  - Df = 293
  - Total sample size = 296
  - Actual power = 0.9505850

# References

1. Armitage CJ, Conner M. Efficacy of the Theory of Planned Behaviour: A meta-analytic review. The British journal of social psychology. 2001;40: 471–499. doi:[10.1348/014466601164939](https://doi.org/10.1348/014466601164939)

2. Rivis A, Sheeran P. Descriptive norms as an additional predictor in the theory of planned behaviour: A meta-analysis. Current Psychology. 2003;22: 218–233. doi:[10.1007/s12144-003-1018-2](https://doi.org/10.1007/s12144-003-1018-2)

3. McEachan RRC, Conner M, Taylor NJ, Lawton RJ. Prospective prediction of health-related behaviours with the Theory of Planned Behaviour: A meta-analysis. Health Psychology Review. 2011;5: 97–144. doi:[10.1080/17437199.2010.521684](https://doi.org/10.1080/17437199.2010.521684)

4. Godin G, Kok G. The theory of planned behavior: A review of its applications to health-related behaviors. American journal of health promotion. 1996;11: 87–98. doi:[10.4278/0890-1171-11.2.87](https://doi.org/10.4278/0890-1171-11.2.87)

5. Downs DS, Hausenblas HA. The Theories of Reasoned Action and Planned Behavior Applied to Exercise: A Meta-analytic Update. Journal of Physical Activity and Health. 2005;2: 76–97. doi:[10.1123/jpah.2.1.76](https://doi.org/10.1123/jpah.2.1.76)

6. Abele-Brehm AE, Gollwitzer M, Steinberg U, Schönbrodt FD. Attitudes Toward Open Science and Public Data Sharing. Social Psychology. 2019;50: 252–260. doi:[10.1027/1864-9335/a000384](https://doi.org/10.1027/1864-9335/a000384)
