# Supplementary material for: Registered report: Survey on attitudes and experiences regarding preregistration in psychological research
Source: PLoS One. 2023 Mar 16;18(3):e0281086. doi: 10.1371/journal.pone.0281086 (PMC10019715; doi:10.1371/journal.pone.0281086)
Supplement: S3 Text — The data collection procedure that is displayed in Fig 2 is further specified. Particularly, the specific procedures for collecting contact addresses as well as for inviting participants are described. (DOCX) [file pone.0281086.s007.docx]

Supporting information to ‘Registered Report: Survey on attitudes and experiences regarding preregistration in psychological research’:

**S7: Data collection procedure**

Lisa Spitzer^1^ & Stefanie Mueller^1^

^1^ Leibniz Institute for Psychology

## Specific procedure for collecting contact addresses

On each website (Web of Science, PubMed, PSYNDEX, PsycInfo, OSF Registries), specific rules were followed for identifying potential participants’ email contacts:

On Web of Science, PubMed, PSYNDEX, and PsycInfo, the search term was set to search “all fields”, it was searched for the keyword “psychology”, and documents were set to articles, to get a broad image of research articles focusing on psychology. Documents were sorted from new to old. All articles that did not focus on psychology were excluded.

On OSF Registries, it was also searched for the keyword “psychology” and documents were sorted from new to old. Only authors of regular preregistrations with focus on psychological research were included. Preregistrations of Registered Reports were excluded, as well as preregistrations of the “Election Research Preacceptance Competition” template as it has no psychological focus, and also other preregistrations that did not focus on psychology. Preregistrations based on the “OSF Standard Pre-Data Collection Preregistration” were also excluded as this is oftentimes an almost empty preregistration. If preregistrations were double or empty, they were not included. Additionally, documents that are clearly no preregistrations were also not considered.

The identified information (source of work, title of work, year, broad topic of work, author name, email address, from which platform was the contact information taken from, link, and comments) was coded in an a priori created coding sheet. Duplicate email addresses that were sampled by different databases were excluded.

## Specific procedure for inviting participants

During data collection, we followed this specific procedure: In a first wave, *N* = 2960 persons whose email addresses were identified as described above were contacted with a personal email. Additionally, the survey was advertised via social media and mailing lists throughout the whole time, pushing the ad every few days. All invited participants were re-contacted after one week with a reminder email. Data collection started on November 15, 2021.

Two weeks after the first invitation (i.e., on November 29, 2021), quotas were checked and for quotas that were not filled yet, more members were recruited. For participants with a master’s degree, a doctoral degree, or a habilitation or full professorship, this was done with the same procedure as described above, yet this time only contact addresses of members of the target quota were collected. The here identified persons were contacted and re-contacted after one week with a reminder. To determine how many people needed to be recruited in the second wave to fill the quotas, we used the response rate from the first invitation wave.

Since we were aiming for 25% quotas, our final sample should contain *n* = 74 participants from each quota (25% of our targeted sample size *N* = 296). After the first invitation wave, only 65 individuals with a habilitation and/or full professorship had participated (for an overview of the quota check, see Table 1), i.e., nine people were still missing in this quota. To calculate how many more participants needed to be invited to fill the quota, the overall response rate of the first invitation wave was used: Since only 268 (instead of 296) of the 2960 invited people had participated (and finished the study), this corresponded to an actual response rate of 9% instead of the estimated 10%. Based on this, we assumed that 9% of invited researchers would also respond in the second invitation wave, thus, 100 more participants would need to be invited to fill our “habilitation and/or professorship” quota (since 9 is 9% of 100). Since we wanted to recruit at the same percentages in all databases as in the first wave (general databases: 22.5% each; OSF: 10%), we invited 102 individuals (23 from each general database, 10 from the OSF). Meanwhile, additional participants with a bachelor’s degree were recruited via social media and mailing lists.

**Table 1. Quota check.**

| **Degree** | **Ideal *n*** | **Quota count after first wave (i.e., only complete datasets)** | **Diff** | ***n* invited in the second wave** | ***N* for hypotheses tests (i.e., only complete datasets)** | ***N* for descriptive reports (i.e., also incomplete datasets)** |
| --- | --- | --- | --- | --- | --- | --- |
| Bachelor’s degree | 74 | 17 | 57 | --- | 38 | 63 |
| Master’s degree | 74 | 85 | --- | 0 | 92 | 124 |
| Doctoral degree | 74 | 101 | --- | 0 | 101 | 161 |
| Habilitation and/or full professorship | 74 | 65 | 9 | 100 | 58 | 72 |

Due to an error, the maximum quota size for the “doctoral degree” quota was exceeded by one person. The final *n* for the “habilitation and/or full professorship” quota for the hypotheses tests is smaller than the *n* after the first invitation wave due to the exclusion of data as specified in the Registered Report. Since the sample for the descriptive reports includes incomplete datasets (which were not considered for the quotas), there are more than 100 participants in some groups. *n* = 52 participants did not answer the “degree” item (however, these are also included in the overall *N* for the descriptive reports).

The second wave started on December 7, 2021, and data collection stopped in accordance with the a priori planned procedure (i.e., two weeks after the second invitation wave). Thus, data collection stopped after participants of the first invitation wave have had the possibility to participate for one month plus one week, and participants of the second invitation wave have had two weeks to participate.
