# Supplementary material for: Registered report: Survey on attitudes and experiences regarding preregistration in psychological research
Source: PLoS One. 2023 Mar 16;18(3):e0281086. doi: 10.1371/journal.pone.0281086 (PMC10019715; doi:10.1371/journal.pone.0281086)
Supplement: S4 Text — Research topics indicated by the sample are displayed in a table. (DOCX) [file pone.0281086.s008.docx]

Supporting information to ‘Registered Report: Survey on attitudes and experiences regarding preregistration in psychological research’:

**S8: Research topics**

Lisa Spitzer^1^ & Stefanie Mueller^1^

^1^ Leibniz Institute for Psychology

**Table 1: Research topics indicated by the sample.**

| **Topic** | **Percentage (Frequency)** |
| --- | --- |
| Clinical psychology | 28.1 (118) |
| Experimental/cognitive psychology | 27.62 (116) |
| Social psychology | 27.38 (115) |
| Research methods | 19.76 (83) |
| Neuroscience/neuropsychology | 19.05 (80) |
| Developmental psychology | 15 (63) |
| Other | 14.05 (59) |
| Educational psychology | 13.57 (57) |
| Organizational psychology | 10.71 (45) |
| General psychology | 9.05 (38) |
| Differential psychology | 8.33 (35) |

Responses given to the item “What is your main research topic (or what is your main interest in psychology)?”, displayed as percentages (and frequencies) of participants who indicated this topic, compared to all participants that responded to the item (*N* = 420). Multiple options could be selected. The option “Psychology is not my main field.” is omitted (participants selecting this option were excluded).

**Table 2: Research topics indicated by the sample (“other” comments).**

| **Topic** | **Percentage (Frequency)** |
| --- | --- |
| Health psychology | 3.1 (13) |
| Personality | 0.95 (4) |
| Environmental psychology | 0.71 (3) |
| Forensic | 0.71 (3) |
| Psychiatry | 0.71 (3) |
| Cross cultural psychology | 0.71 (3) |
| School psychology | 0.48 (2) |
| Traffic/engineering psychology | 0.48 (2) |
| Political psychology | 0.48 (2) |
| Meta-research | 0.48 (2) |
| Evolutionary psychology | 0.48 (2) |
| Human-computer-interaction | 0.48 (2) |
| Consumer psychology | 0.24 (1) |
| Quantitative psychology | 0.24 (1) |
| Computational social science | 0.24 (1) |
| Socio-cultural psychology | 0.24 (1) |
| Animal-assisted interventions | 0.24 (1) |
| Applied developmental psychology | 0.24 (1) |
| Sport psychology | 0.24 (1) |
| Abnormal psychology | 0.24 (1) |
| Behavior analysis | 0.24 (1) |
| Industrial/organizational psychology | 0.24 (1) |
| Psychological assessment | 0.24 (1) |
| Psycho-oncology | 0.24 (1) |
| Affective science | 0.24 (1) |
| Special education | 0.24 (1) |
| Psycholinguistics | 0.24 (1) |
| Community psychology | 0.24 (1) |
| Positive psychology | 0.24 (1) |

“Other” comments given to the item “What is your main research topic (or what is your main interest in psychology)?”, displayed as percentages (and frequencies) of participants who indicated this topic, compared to all participants that responded to the item (*N* = 420).
