# Supplementary material for: Registered report: Survey on attitudes and experiences regarding preregistration in psychological research
Source: PLoS One. 2023 Mar 16;18(3):e0281086. doi: 10.1371/journal.pone.0281086 (PMC10019715; doi:10.1371/journal.pone.0281086)
Supplement: S5 Text — All exploratory analyses and results are described in this document. (DOCX) [file pone.0281086.s009.docx]

Supporting information to ‘Registered Report: Survey on attitudes and experiences regarding preregistration in psychological research’:

**S13: Exploratory analyses**

Lisa Spitzer^1^ & Stefanie Mueller^1^

^1^ Leibniz Institute for Psychology

# Exploratory analyses

In addition to our planned analyses, various exploratory analyses were conducted, which are described in the following. Some of these analyses tested for significance, and Bonferroni-Holm correction was applied whenever multiple tests were conducted. However, since these are exploratory results, *p* values should be interpreted with caution. The code for all exploratory analyses is provided in S12 Script.

## Influence of staying in or leaving academia

First, we conducted additional sensitivity analyses to determine if controlling whether subjects wanted to stay in or leave academia would have an impact on the results. It is conceivable that participants who, for example, are disappointed with working in research and want to leave academia, may have a different opinion of preregistration or are more likely not to intend to use preregistration in the future. The results of these sensitivity analyses indicated that wanting to stay in or leave academia did not change the results of the hypotheses tests reported above, nor was it a significant predictor for intention, attitudes, or perceived motivations and obstacles (all *p_cor_* > .05).

## Participants recruited via different databases

We recruited participants from different databases, e-mail lists, and social media. Table 1 displays participants’ average scores on the scales implemented in the study, by the database they were recruited from. As may be expected, the mean values of the participants recruited via OSF Registries, i.e., a preregistration database, are above those of the other recruitment sources, meaning that participants from OSF responded more favorably regarding preregistration.

**Table 1. Differences between participants recruited from different sources.**

|  | **Web of Science** | **PubMed** | **PSYNDEX** | **PsycInfo** | **OSF** | **E-Mail** | **Social Media** |
| --- | --- | --- | --- | --- | --- | --- | --- |
| Attitude scale | 0.78 (1.07)  *range* = 4.79 | 0.83 (0.99)  *range* = 4.13 | 0.97 (1.08)  *range* = 4.63 | 0.83 (1.15)  *range* = 4.04 | 1.53 (0.81)  *range* = 3.42 | 1.45 (0.67)  *range* = 2.92 | 0.94 (0.93)  *range* = 4.21 |
| Motivation scale | 0.27 (1.01)  *range* = 4.2 | 0.54 (0.8)  *range* = 3.4 | 0.5 (1.11)  *range* = 5.3 | 0.4 (1.01)  *range* = 4 | 1.1 (0.83)  *range* = 3.6 | 0.87 (0.72)  *range* = 3.7 | 0.85 (0.75)  *range* = 2.9 |
| Obstacle scale | -0.28 (1.19)  *range* = 5.3 | -0.33 (1.29)  *range* = 4.5 | -0.33 (1.02)  *range* = 4.8 | -0.14 (1.06)  *range* = 4.3 | -1.03 (0.81)  *range* = 3.2 | -0.52 (0.99)  *range* = 4.2 | -0.2 (1.22)  *range* = 4.7 |
| Subjective norm scale | 0.21 (0.68)  *range* = 2.63 | 0.38 (0.87)  *range* = 3.38 | 0.36 (0.96)  *range* = 4.13 | 0.44 (0.78)  *range* = 2.75 | 1.02 (0.7)  *range* = 2.88 | 0.63 (0.87)  *range* = 3.88 | 0.52 (0.86)  *range* = 4.13 |
| Perceived behavioral control scale | 0.87 (1.04)  *range* = 3.8 | 1 (1.17)  *range* = 4.6 | 1.19 (1.09)  *range* = 4.4 | 0.77 (1.29)  *range* = 3.8 | 1.66 (0.88)  *range* = 3.2 | 0.96 (1.29)  *range* = 4.8 | 0.5 (1.11)  *range* = 4.6 |
| Intention scale | 1.22 (1.66)  *range* = 6 | 1.19 (1.41)  *range* = 5.67 | 1.26 (1.63)  *range* = 6 | 1.17 (1.45)  *range* = 5 | 2.34 (0.82)  *range* = 3 | 1.86 (0.97)  *range* = 3.67 | 1.23 (1.26)  *range* = 6 |
| Importance | 0.44 (1.7)  *range* = 6 | 0.53 (1.61)  *range* = 6 | 0.67 (1.77)  *range* = 6 | 0.33 (1.81)  *range* = 6 | 1.67 (1.66)  *range* = 6 | 1.46 (1.2)  *range* = 5 | 1.27 (1.46)  *range* = 5 |

The following parameters are displayed: *Mean* (*SD*), *range* for the scales; percentage of indication “yes” for preregistration experience. Scales ranged from -3 to 3. These parameters were calculated using the sample for the hypotheses tests (i.e., only complete datasets, *N* = 289).

## Gender differences

Next, we were interested in gender differences. The descriptive results are displayed in Table 2. Participants that indicated “other gender” yielded higher average scores in all scales except for the obstacle scale, which means that they had more positive attitudes, perceived motivations more strongly, experienced a high subjective norm and high perceived behavioral control, had a high intention to preregister, and found preregistration important, whereas obstacles were experienced less strongly. All of these participants had preregistered before. Women yielded higher average scores than men in all scales except the perceived behavioral control and obstacle scales, that is, they felt less control over the behavior, but at the same time, experienced obstacles less strongly. Preregistration experience was similar between male and female participants, with a slightly higher proportion of men with preregistration experience. Means of male and female participants were compared with Welch *t*-tests, but no significant *p* values were obtained after Bonferroni-Holm correction (all *p_cor_* > .05). No *t*-test was conducted for participants that indicated “other gender” because of their small group size.

**Table 2. Gender differences per scale and preregistration experience.**

|  | **Male**  ***n* = 122** | **Female**  **n = 153** | **Other gender**  ***n* = 3** |
| --- | --- | --- | --- |
| Attitude scale | 0.99 (0.97)  *range* = 4.79 | 1.27 (0.94)  *range* = 4.67 | 1.6 (0.78)  *range* = 1.42 |
| Motivation scale | 0.59 (0.78)  *range* = 4.5 | 0.82 (1.02)  *range* = 5.4 | 1.03 (0.84)  *range* = 1.5 |
| Obstacle scale | -0.31 (1.05)  *range* = 4.8 | -0.54 (1.12)  *range* = 5.6 | -1.63 (0.97)  *range* = 1.9 |
| Subjective norm scale | 0.46 (0.8)  *range* = 3.62 | 0.6 (0.9)  *range* = 4.38 | 1.75 (0.5)  *range* = 1 |
| Perceived behavioral control scale | 1.15 (1.04)  *range* = 4.6 | 0.93 (1.24)  *range* = 4.8 | 2 (0)  *range* = 0 |
| Intention scale | 1.45 (1.34)  *range* = 6 | 1.64 (1.37)  *range* = 6 | 2.89 (0.19)  *range* = 0.33 |
| Importance | 0.9 (1.58)  *range* = 6 | 1.21 (1.63)  *range* = 6 | 2.33 (1.15)  *range* = 2 |
| Preregistration experience - yes | 68.65% of 134 responses | 64.24% of 179 responses | 100% of 3 responses |

The following parameters are displayed: *Mean* (*SD*), *range* for the scales; percentage of indication “yes” for preregistration experience. Scales ranged from -3 to 3. While the parameters concerning the scales were calculated using the sample for the hypotheses tests (i.e., only complete datasets), the preregistration experience is displayed for all participants that answered this item.

## Influence of age

Moreover, we wanted to explore the influence of age on our scales. Age was correlated highly with research experience (*r* = .928, *t*(285) = 41.89, *p_cor_* < .001). Furthermore, age was also associated with attitudes (*r* = -.266, *t*(285) = -4.65, *p_cor_* < .001), motivations (*r* = -.256, *t*(285) = -4.47, *p_cor_* < .001), intention (*r* = -.195, *t*(285) = -3.36, *p_cor_* = .002), and importance (*r* = -.214, *t*(285) = -3.69, *p_cor_* < .001), but not with the perception of obstacles (*r* = .051, *t*(285) = 0.85, *p_cor_* = .394). This shows that the younger the researchers were, the more positive their attitudes were, the stronger they perceived motivations to preregister, the more they intended to use preregistration, and the more important they found it. Meanwhile, obstacles were perceived the same regardless of age.

## Correlation of attitudes, motivations, and obstacles

We were also interested in the direct relationship between researchers’ attitudes and perceived motivations and obstacles. It can be assumed that attitudes and motivations are positively associated, while both are negatively correlated with perceived obstacles. And indeed, this pattern was identified in the data: While attitudes and motivations showed a strong positive correlation (*r* = .781, *t*(287) = 21.21, *p_cor_* < .001), both correlated negatively with perceived obstacles (attitudes – obstacles: *r* = -.558, *t*(287) = -11.38, *p_cor_* < .001; motivations – obstacles: *r* = -.436, *t*(287) = ‑8.21, *p_cor_* < .001).

## Worries and problems for different research topics and degrees

It is conceivable that the reported worries and problems differ between the various research topics and academic groups. Thus, an overview of the percentage of participants in each research topic or academic group who reported the respective worries and problems can be found in the Tables 3 and 4.

**Table 3. Worries and problems indicated by participants with different research topics.**

| **Research topic** |  | **Insecurity** | **Conflict** | **Changes** | **Errors** | **Credibility** | **Flexibility** | **Scooping** | **Time** |
| --- | --- | --- | --- | --- | --- | --- | --- | --- | --- |
| Clinical | Worry  (*N* = 31) | 38.71 | 6.45 | 48.39 | 22.58 | 35.48 | 48.39 | 41.94 | 67.74 |
|  | Problem  (*N* = 39) | 43.59 | 10.26 | 15.38 | 15.38 | 23.08 | 17.95 | 7.69 | 56.41 |
| Developmental | Worry  (*N* = 15) | 40 | 20 | 40 | 46.67 | 40 | 33.33 | 26.67 | 53.33 |
|  | Problem  (*N* = 29) | 41.38 | 3.45 | 20.69 | 10.34 | 10.34 | 20.69 | 0 | 62.07 |
| Differential | Worry  (*N* = 7) | 42.86 | 14.29 | 71.43 | 57.14 | 28.57 | 42.86 | 57.14 | 57.14 |
|  | Problem  (*N* = 16) | 43.75 | 0 | 12.5 | 25 | 25 | 6.25 | 0 | 43.75 |
| Educational | Worry  (*N* = 12) | 33.33 | 25 | 41.67 | 41.67 | 33.33 | 58.33 | 33.33 | 33.33 |
|  | Problem  (*N* = 27) | 33.33 | 3.7 | 14.81 | 14.81 | 18.52 | 25.93 | 7.41 | 44.44 |
| Experimental / cognitive | Worry  (*N* = 31) | 32.26 | 16.13 | 38.71 | 32.26 | 48.39 | 58.06 | 41.94 | 61.29 |
|  | Problem  (*N* = 58) | 44.83 | 6.9 | 10.34 | 36.21 | 12.07 | 22.41 | 1.72 | 51.72 |
| General | Worry  (*N* = 15) | 20 | 26.67 | 53.33 | 46.67 | 26.67 | 73.33 | 46.67 | 60 |
|  | Problem  (*N* = 11) | 27.27 | 9.09 | 36.36 | 27.27 | 27.27 | 18.18 | 27.27 | 45.45 |
| Neuro | Worry  (*N* = 27) | 25.93 | 18.52 | 37.04 | 22.22 | 44.44 | 44.44 | 33.33 | 66.67 |
|  | Problem  (*N* = 33) | 42.42 | 12.12 | 15.15 | 21.21 | 24.24 | 18.18 | 9.09 | 54.55 |
| Organizational | Worry  (*N* = 7) | 28.57 | 14.29 | 42.86 | 28.57 | 71.43 | 42.86 | 57.14 | 42.86 |
|  | Problem  (*N* = 23) | 39.13 | 8.7 | 8.7 | 34.78 | 30.43 | 0 | 4.35 | 30.43 |
| Methods | Worry  (*N* = 17) | 11.76 | 11.76 | 64.71 | 17.65 | 41.18 | 64.71 | 58.82 | 58.82 |
|  | Problem  (*N* = 49) | 38.78 | 4.08 | 6.12 | 28.57 | 10.2 | 16.33 | 6.12 | 44.9 |
| Social | Worry  (*N* = 18) | 16.67 | 22.22 | 44.44 | 22.22 | 61.11 | 72.22 | 33.33 | 50 |
|  | Problem  (*N* = 59) | 55.93 | 8.47 | 13.56 | 23.73 | 16.95 | 15.25 | 3.39 | 33.9 |

For each research topic, the percentage of participants that indicated a worry / problem in comparison to all participants that answered the respective item is displayed. See S2 Table for a list of complete response options (abbreviated here, e.g., “conflict”). For brevity, the options “none” and “other” were omitted in this table.

**Table 4. Worries and problems indicated by participants with different degrees.**

| **Degree** |  | **Insecurity** | **Conflict** | **Changes** | **Errors** | **Credibility** | **Flexibility** | **Scooping** | **Time** |
| --- | --- | --- | --- | --- | --- | --- | --- | --- | --- |
| Bachelor’s | Worry  (*N* = 20) | 40 | 30 | 55 | 40 | 25 | 50 | 45 | 55 |
|  | Problem  (*N* = 18) | 55.56 | 11.11 | 16.67 | 27.78 | 16.67 | 27.78 | 16.67 | 27.78 |
| Master’s | Worry  (*N* = 30) | 30 | 20 | 36.67 | 20 | 46.67 | 43.33 | 30 | 53.33 |
|  | Problem  (*N* = 57) | 49.12 | 12.28 | 17.54 | 24.56 | 21.05 | 14.04 | 3.51 | 43.86 |
| Doctoral | Worry  (*N* = 30) | 26.67 | 6.67 | 46.67 | 23.33 | 56.67 | 63.33 | 33.33 | 70 |
|  | Problem  (*N* = 71) | 40.85 | 7.04 | 9.86 | 26.76 | 15.49 | 14.08 | 2.82 | 46.48 |
| Habilitation / full professorship | Worry  (*N* = 14) | 21.43 | 0 | 57.14 | 28.57 | 42.86 | 64.29 | 50 | 71.43 |
|  | Problem  (*N* = 44) | 25 | 9.09 | 13.64 | 22.73 | 15.91 | 13.64 | 4.55 | 52.27 |

For each degree, the percentage of participants that indicated a worry / problem in comparison to all participants that answered the respective item is displayed. See S2 Table for a list of complete response options (abbreviated here, e.g., “conflict”). For brevity, the options “none” and “other” were omitted in this table.

## Scooping and intention to preregister

Since *n* = 9 participants indicated having been scooped, we investigated if this influenced their perception of preregistration. Descriptively, their intention, attitudes and motivations seemed to be decreased, while the perceived obstacles were indicated with a higher average (see Table 5). Because group sizes varied considerably, no significance tests were performed. Despite having been scooped, six of the scooped participants were inclined to preregistering their next study (i.e., their intention score was greater than zero).

**Table 5. Perception of preregistration by scooped researchers.**

|  | **Not scooped**  ***N* = 189** | **Scooped**  ***N* = 9** |
| --- | --- | --- |
| Intention scale | 1.93 (1.15)  *range* = 5 | 0.96 (1.2)  *range* = 3.67 |
| Attitude scale | 1.27 (0.93)  *range* = 4.67 | 0.44 (0.95)  *range* = 3.38 |
| Motivation scale | 0.85 (0.87)  *range* = 5.3 | 0.11 (0.75)  *range* = 2.3 |
| Obstacle scale | -0.72 (1.03)  *range* = 4.5 | 0.09 (0.71)  *range* = 2.5 |

The following parameters are displayed: *Mean* (*SD*), *range*. Scales ranged from -3 to 3. When interpreting these parameters, it must be noted that the group of participants that was scooped was very small.

## Participants with preregistration experience that do not want to preregister again

Finally, we looked more closely at researchers with preregistration experience who do not intend to preregister again, to identify negative preregistration experiences they may have had. Twelve participants who had previously preregistered were undecided about preregistering again (i.e., their intention score equaled 0). Meanwhile, ten participants tended not wanting to preregister again (i.e., their intention score was less than 0). From these, eight participants planned to continue their careers in research. Again, no significance tests were conducted due to the small group size. Descriptively, participants who had preregistered before but did not intend to do so again had lower scores for the intention, attitude, and motivation scale, while their mean score for the obstacle scale was higher than that of participants with preregistration experience who planned to preregister again or were indifferent toward it (see Table 6).

**Table 6. Perception of preregistration by researchers with preregistration experience who do not intend to preregister again.**

|  | **Intend to preregister**  **again / are indifferent (intention score ≥ 0)**  ***N* = 202** | **Intend not to**  **preregister again**  **(intention score < 0)**  ***N* = 10** |
| --- | --- | --- |
| Intention scale | 1.58 (1.31)  *range* = 6 | -1 (0.59)  *range* =1.67 |
| Attitude scale | 1.08 (0.97)  *range* = 5.51 | -0.25 (0.72)  *range* = 2.71 |
| Motivation scale | 0.73 (0.89)  *range* = 5.4 | -0.67 (0.99)  *range* = 3 |
| Obstacle scale | -0.43 (1.09)  *range* = 5.6 | 0.11 (0.79)  *range* = 3.2 |

The following parameters are displayed: *Mean* (*SD*), *range*. Scales ranged from -3 to 3. When interpreting these parameters, it must be noted that the group of participants with preregistration experience who did not intend to preregister again was very small.

Visual inspection of the data did not reveal any patterns explaining why ten these participants would not want to preregister again, other than for general reasons such as effort and time. When inspecting these participants’ responses to the item “Did you encounter specific problems when preregistering a study? If yes, which ones?” more closely, around a third (30%) had experienced conflicts with their supervisors or co-authors, reported that deviations were necessary and their study lost credibility, or indicated that they found it problematic to not have flexibility during the analyses. Twenty percent reported that the study design would have needed to be changed which was not possible, or that it took very long to do the preregistration. Ten percent were insecure about what needed to be included, reported that errors in the preregistration could not be changed afterwards, or that they were scooped. It might be the case that the problems reported with a higher frequency here than in the general sample of researchers with preregistration experience (e.g., conflict or lost credibility) might have led to these researchers not wanting to preregister again, and it might be interesting to investigate this further in future studies.
