# Supplementary material for: Registered report: Survey on attitudes and experiences regarding preregistration in psychological research
Source: PLoS One. 2023 Mar 16;18(3):e0281086. doi: 10.1371/journal.pone.0281086 (PMC10019715; doi:10.1371/journal.pone.0281086)
Supplement: S6 Text — Participants’ suggestions for improving the preregistration process are displayed in a table. (DOCX) [file pone.0281086.s010.docx]

Supporting information to ‘Registered Report: Survey on attitudes and experiences regarding preregistration in psychological research’:

**S14: Suggestions for improvements regarding the preregistration process**

Lisa Spitzer^1^ & Stefanie Mueller^1^

^1^ Leibniz Institute for Psychology

**Table 1. Suggestions for improvements regarding the preregistration process.**

| **Improvements regarding templates** | **With PE**  ***n* = 42** | **Without PE *n* = 13** | **Overall**  ***N* = 55** |
| --- | --- | --- | --- |
| Easy usability | 11.9 (5) | 38.46 (5) | 18.18 (10) |
| Clear guidelines / more standardization | 11.9 (5) | 30.77 (4) | 16.36 (9) |
| Good examples | 11.9 (5) | 15.38 (2) | 12.73 (7) |
| More automated process with many possibilities (e.g., including tables and figures) | 11.9 (5) | 7.69 (1) | 10.91 (6) |
| Appropriate set of template types (fitting different research situations) | 11.9 (5) | 0 (0) | 9.09 (5) |
| Templates with multiple levels of complexity (e.g., short template where extra items can be added depending on study, “other” options) | 7.14 (3) | 0 (0) | 5.45 (3) |
| Much flexibility (e.g., “not yet planned” option) | 7.14 (3) | 0 (0) | 5.45 (3) |
| **Repositories and publication** | **With PE**  ***n* = 32** | **Without PE *n* = 10** | **Overall**  ***N* = 42** |
| Good platforms (user-friendly, integration of various existing platforms) | 46.88 (15) | 50 (5) | 47.62 (20) |
| Enable easy tracking of changes (e.g., allow for timestamped revisions) | 12.5 (4) | 10 (1) | 11.9 (5) |
| **Review process** | **With PE**  **(*n* = 37)** | **Without PE (*n* = 10)** | **Overall**  **(*N* = 47)** |
| Make it quick | 8.11 (3) | 30 (3) | 12.77 (6) |
| Accept deviations | 10.81 (4) | 10 (1) | 10.64 (5) |
| Check preregistration against manuscript | 13.51 (5) | 0 (0) | 10.64 (5) |
| Positively consider null results | 8.11 (3) | 0 (0) | 6.38 (3) |
| Detailed, easily addressable feedback | 5.41 (2) | 10 (1) | 6.38 (3) |
| **Integration of preregistrations in published articles** | **With PE**  ***n* = 32** | **Without PE *n* = 7** | **Overall**  ***N* = 39** |
| Make it mandatory | 25 (8) | 14.29 (1) | 23.08 (9) |
| Include link to preregistration | 25 (8) | 14.29 (1) | 23.08 (9) |
| Badges | 15.63 (5) | 14.29 (1) | 15.38 (6) |
| Nothing | 6.25 (2) | 14.29 (1) | 7.69 (3) |
| Clear reporting of deviations | 6.25 (2) | 14.29 (1) | 7.69 (3) |
| Guidelines | 6.25 (2) | 0 (0) | 5.13 (2) |
| Make it more obvious what was preregistered | 6.25 (2) | 0 (0) | 5.13 (2) |

| **Education** | **With PE**  ***n* = 38** | **Without PE *n* = 14** | **Overall**  ***N* = 52** |
| --- | --- | --- | --- |
| Incorporation in study curricula | 36.84 (14) | 35.71 (5) | 36.54 (19) |
| Mandatory courses / workshops | 34.21 (13) | 35.71 (5) | 34.62 (18) |
| Improve accessibility | 5.26 (2) | 7.14 (1) | 5.77 (3) |
| Examples / templates | 5.26 (2) | 7.14 (1) | 5.77 (3) |
| Guidance on how to deal with deviations | 7.89 (3) | 0 (0) | 5.77 (3) |

Responses are displayed as percentages of participants who indicated a theme compared to all participants with or without preregistration experience (= PE) that responded to the item. Absolute numbers of participants that indicated a theme are displayed in parentheses. For brevity, only themes indicated by more than 5% of the overall sample are displayed.
