# Supplementary material for: Registered report: Survey on attitudes and experiences regarding preregistration in psychological research
Source: PLoS One. 2023 Mar 16;18(3):e0281086. doi: 10.1371/journal.pone.0281086 (PMC10019715; doi:10.1371/journal.pone.0281086)
Supplement: S7 Text — Tables containing information about the individual survey items (means, standard deviations, and distribution of responses) are provided here for both the general and the database sample (i.e., only including participants recruited via the databases). (DOCX) [file pone.0281086.s011.docx]

Supporting information to ‘Registered Report: Survey on attitudes and experiences regarding preregistration in psychological research’:

**S15: Overview of survey items**

Lisa Spitzer^1^ & Stefanie Mueller^1^

^1^ Leibniz Institute for Psychology

**Table 1. Overview of survey items (sample used for the hypotheses test, also including participants recruited from the OSF).**

|  |  | **Statistics** | | **Distribution of responses (%)** | | | | | | |
| --- | --- | --- | --- | --- | --- | --- | --- | --- | --- | --- |
| **Item** | **Text** | ***M*** | ***SD*** | **-3** | **-2** | **-1** | **0** | **1** | **2** | **3** |
| A1 | I have more trust in research findings when the study has been preregistered. | 1.19 | 1.51 | 2.77 | 5.19 | 4.5 | 13.84 | 24.57 | 29.41 | 19.72 |
| A2 | I have more trust in researchers who preregister their studies than in those who don’t. | 1.05 | 1.6 | 3.81 | 6.92 | 4.15 | 13.84 | 26.64 | 25.95 | 18.69 |
| A3 | Science profits from preregistration. | 1.58 | 1.36 | 1.73 | 2.08 | 2.42 | 12.8 | 21.8 | 29.41 | 29.76 |
| A4 | Preregistration of studies should be obligatory. | 0.16 | 1.77 | 10.73 | 10.38 | 12.11 | 19.03 | 22.15 | 17.65 | 7.96 |
| A5 | In selection decisions people who preregister their studies should be preferred (given the same qualification). | 0.51 | 1.81 | 9.34 | 8.3 | 9.34 | 15.92 | 22.15 | 21.8 | 13.15 |
| A6 | Some researchers exploit preregistration (e.g., by cheating).* | -0.01 | 1.33 | 2.77 | 15.22 | 9.34 | 39.79 | 20.42 | 10.38 | 2.08 |
| A7 | I think that preregistration does not improve research significantly.* | -1.29 | 1.5 | 23.53 | 30.8 | 19.03 | 12.46 | 7.61 | 5.54 | 1.04 |
| A8 | Preregistration cannot prevent questionable research practices.* | 0.49 | 1.56 | 1.73 | 10.73 | 18.69 | 13.15 | 25.61 | 21.11 | 9 |
| A9 | Preregistration can prevent selective reporting (i.e., only reporting variables that yielded significant results). | 1.36 | 1.36 | 1.38 | 3.11 | 6.92 | 10.03 | 21.11 | 40.14 | 17.3 |
| A10 | Preregistration can prevent p-hacking (i.e., misusing data analyses to find patterns that can be presented as statistically significant). | 1.1 | 1.49 | 1.73 | 5.88 | 9 | 10.38 | 26.3 | 30.8 | 15.92 |
| A11 | Preregistration can prevent publication bias (i.e., only publishing positive/significant results). | 0.81 | 1.68 | 4.15 | 9.69 | 7.61 | 13.84 | 23.53 | 26.3 | 14.88 |
| A12 | Preregistration improves a study’s quality. | 1 | 1.48 | 2.42 | 5.54 | 7.61 | 13.84 | 29.76 | 25.95 | 14.88 |
| A13 | Preregistration increases the credibility of psychological research. | 1.46 | 1.36 | 1.04 | 4.15 | 4.84 | 7.61 | 24.91 | 34.95 | 22.49 |
| A14 | Preregistration is a bad initiative.* | -2.07 | 1.34 | 54.33 | 22.15 | 8.65 | 9.34 | 2.77 | 1.73 | 1.04 |
| A15 | The costs of preregistering a study are higher than the usefulness of preregistering it.* | -0.99 | 1.61 | 21.8 | 21.8 | 18.34 | 20.76 | 8.65 | 6.57 | 2.08 |
| A16 | Preregistering studies is generally unnecessary.* | -1.53 | 1.43 | 30.1 | 29.07 | 20.76 | 10.73 | 3.46 | 5.19 | 0.69 |
| A17 | Science should be open and transparent. | 2.44 | 0.98 | 0 | 0.69 | 2.42 | 3.11 | 5.19 | 23.18 | 65.4 |
| A18 | I feel that nowadays, a great variety of new structures help creating preregistrations, for example templates or repositories (i.e., uploading platforms). | 1.17 | 1.31 | 1.04 | 3.11 | 3.81 | 23.53 | 21.45 | 32.87 | 14.19 |
| A19 | Preregistration makes science more transparent. | 1.77 | 1.28 | 1.38 | 2.08 | 2.77 | 6.57 | 17.65 | 38.41 | 31.14 |
| A20 | Preregistration is very useful. | 1.38 | 1.43 | 1.38 | 4.84 | 4.84 | 10.03 | 23.53 | 33.22 | 22.15 |
| A21 | Preregistration is not useful in practice.* | -1.59 | 1.43 | 32.53 | 30.1 | 16.26 | 10.73 | 6.92 | 2.08 | 1.38 |
| A22 | Preregistration hinders exploratory research.* | -0.43 | 1.87 | 18.69 | 18.34 | 11.07 | 13.15 | 21.45 | 12.8 | 4.5 |
| A23 | Preregistration decreases scientific progress.* | -1.46 | 1.57 | 31.83 | 28.72 | 14.19 | 11.76 | 7.96 | 3.11 | 2.42 |
| A24 | A preregistration badge (i.e., a public acknowledgment that a study was preregistered provided by many journals) increases my trust in a study. | 0.82 | 1.54 | 3.81 | 8.3 | 4.84 | 15.92 | 29.41 | 27.34 | 10.38 |
| SN1 | My peers and colleagues motivate me to preregister my studies. | 0.52 | 1.61 | 5.19 | 10.38 | 5.88 | 24.22 | 23.53 | 22.15 | 8.65 |
| SN2 | I want to be part of the open science movement. | 1.56 | 1.36 | 2.08 | 1.73 | 2.42 | 12.8 | 22.49 | 30.1 | 28.37 |
| SN3 | My co-authors and supervisors want me to preregister my studies. | 0.3 | 1.67 | 5.54 | 13.49 | 8.3 | 28.03 | 16.96 | 17.99 | 9.69 |
| SN4 | Preregistration is highly acknowledged in psychological science. | 0.57 | 1.31 | 2.77 | 3.11 | 15.57 | 19.38 | 35.29 | 19.72 | 4.15 |
| SN5 | In today's psychological research community, it is deemed necessary to preregister. | 0.17 | 1.35 | 1.38 | 12.46 | 17.65 | 23.18 | 28.72 | 14.88 | 1.73 |
| SN6 | I feel social pressure to preregister my studies. | -0.34 | 1.65 | 10.03 | 20.76 | 14.88 | 17.99 | 23.18 | 9.69 | 3.46 |
| SN7 | I feel social pressure not to preregister my studies.* | -1.69 | 1.49 | 41.18 | 25.26 | 10.73 | 11.76 | 7.27 | 3.11 | 0.69 |
| SN8 | I think that many researchers preregister their studies. | -0.18 | 1.36 | 2.42 | 15.92 | 24.91 | 24.91 | 19.72 | 10.38 | 1.73 |
| PBC1 | It would be easy for me to preregister my studies. | 0.72 | 1.6 | 1.73 | 8.65 | 15.92 | 14.19 | 22.84 | 22.84 | 13.84 |
| PBC2 | I know how to create and upload a preregistration. | 1.28 | 1.9 | 4.84 | 9.69 | 7.96 | 3.46 | 13.49 | 25.26 | 35.29 |
| PBC3 | With the current implementation of preregistration, it is my decision if I want to preregister my studies. | 1.49 | 1.35 | 1.73 | 3.46 | 3.46 | 9 | 20.42 | 41.18 | 20.76 |
| PBC4 | Even if I wanted to preregister, there are external factors that hinder me (e.g., my co-authors or supervisors do not want to preregister).* | -0.94 | 1.73 | 22.49 | 26.99 | 9 | 17.3 | 15.57 | 5.19 | 3.46 |
| PBC5 | I don’t feel well informed about preregistration.* | -0.63 | 1.88 | 18.34 | 25.61 | 11.76 | 13.84 | 12.11 | 12.46 | 5.88 |
| I1 | I will preregister my studies in the near future. | 1.48 | 1.47 | 2.08 | 3.11 | 4.15 | 12.46 | 21.45 | 26.64 | 30.1 |
| I2 | I intend to preregister my studies in the future. | 1.53 | 1.43 | 2.08 | 2.77 | 2.42 | 13.49 | 22.49 | 25.61 | 31.14 |
| I3 | I want to use preregistration in the future. | 1.58 | 1.47 | 2.42 | 2.77 | 3.81 | 10.73 | 19.03 | 28.37 | 32.87 |
| M1 | I feel like preregistration is an investment in my future (e.g., it is helpful for my career). | 0.64 | 1.54 | 3.46 | 7.96 | 7.61 | 25.95 | 24.22 | 18.69 | 12.11 |
| M2 | I have the feeling that nowadays, it is harder to publish studies that were not preregistered. | -0.12 | 1.43 | 3.46 | 15.57 | 20.42 | 28.03 | 17.99 | 11.42 | 3.11 |
| M3 | Preregistration helps me to plan my study in more detail. | 1.54 | 1.41 | 1.73 | 3.81 | 2.08 | 10.38 | 24.91 | 27.68 | 29.41 |
| M4 | The preregistration badge (i.e., a public acknowledgment that a study was preregistered provided by many journals) would be an incentive for me to preregister my studies. | 0.23 | 1.62 | 6.92 | 13.49 | 7.61 | 20.76 | 29.41 | 16.61 | 5.19 |
| M5 | I feel morally obligated to preregister my studies. | 0.4 | 1.77 | 8.65 | 10.03 | 9.34 | 17.3 | 25.61 | 17.3 | 11.76 |
| M6 | I think planning studies in detail in the context of preregistration is fun. | 0.47 | 1.61 | 5.54 | 8.3 | 11.76 | 20.42 | 24.57 | 20.76 | 8.65 |
| M7 | Preregistration helps making my studies more transparent. | 1.73 | 1.26 | 1.38 | 1.73 | 2.42 | 7.96 | 19.38 | 37.72 | 29.41 |
| M8 | I want others to be able to comment on my planned studies. | 0.62 | 1.57 | 3.46 | 9.34 | 9.34 | 20.76 | 23.53 | 23.88 | 9.69 |
| M9 | It is necessary that preregistration limits my flexibility. | 0.24 | 1.55 | 5.88 | 9.34 | 13.84 | 24.57 | 24.57 | 15.92 | 5.88 |
| M10 | I use preregistration because it represents good scientific practice for me. | 1.28 | 1.58 | 2.77 | 4.84 | 3.11 | 19.72 | 17.3 | 24.57 | 27.68 |
| O1 | I am afraid that I could have a competitive disadvantage when I preregister my studies. | -0.96 | 1.72 | 21.8 | 24.57 | 17.3 | 14.19 | 12.11 | 5.88 | 4.15 |
| O2 | Given the present scientific context, preregistering my studies hinders my career. | -1.22 | 1.58 | 26.99 | 25.26 | 14.88 | 16.26 | 10.73 | 4.5 | 1.38 |
| O3 | I am afraid that after preregistering my studies others will find errors or deviations in/from my study plans. | -0.39 | 1.67 | 11.07 | 23.88 | 10.73 | 16.61 | 24.91 | 10.73 | 2.08 |
| O4 | I don't like that preregistration limits my flexibility. | -0.28 | 1.82 | 13.49 | 19.03 | 13.84 | 14.19 | 21.11 | 12.11 | 6.23 |
| O5 | Preregistration causes considerable time cost. | 0.72 | 1.61 | 4.5 | 9 | 7.96 | 13.49 | 29.76 | 24.22 | 11.07 |
| O6 | For me, there are not enough incentives to preregister my studies. | -0.48 | 1.71 | 15.57 | 17.3 | 16.61 | 17.3 | 19.03 | 11.42 | 2.77 |
| O7 | I would be afraid of scooping (i.e., someone taking my idea and publishing it before me) when preregistering my study. | -0.47 | 1.83 | 14.88 | 23.88 | 13.84 | 10.38 | 18.34 | 14.88 | 3.81 |
| O8 | I am unsure about confidentiality issues and intellectual property rights when preregistering my studies. | -0.16 | 1.78 | 12.46 | 16.61 | 10.73 | 20.07 | 21.11 | 12.46 | 6.57 |
| O9 | For my projects, preregistration is unnecessary. | -0.75 | 1.74 | 20.07 | 22.15 | 11.07 | 22.15 | 12.46 | 8.65 | 3.46 |
| O10 | Preregistration slows down the scientific progress of my project. | -0.33 | 1.7 | 11.76 | 17.99 | 17.65 | 15.92 | 21.11 | 11.76 | 3.81 |

Means, standard deviations, and distribution of responses for all scale items, based on the data of the sample that was used for the hypotheses tests (i.e., complete datasets, *N* = 289). Items marked with * are negatively poled and were reversed for further analysis. -3 = Strongly disagree. 3 = Strongly agree.

**Table 2. Overview of survey items (sample used for the hypotheses test which was recruited via the databases).**

|  |  | **Statistics** | | **Distribution of responses (%)** | | | | | | |
| --- | --- | --- | --- | --- | --- | --- | --- | --- | --- | --- |
| **Item** | **Text** | ***M*** | ***SD*** | **-3** | **-2** | **-1** | **0** | **1** | **2** | **3** |
| A1 | I have more trust in research findings when the study has been preregistered. | 1.05 | 1.72 | 4.55 | 9.09 | 2.27 | 15.91 | 18.94 | 28.03 | 21.21 |
| A2 | I have more trust in researchers who preregister their studies than in those who don’t. | 0.59 | 1.86 | 7.58 | 12.88 | 4.55 | 17.42 | 18.94 | 22.73 | 15.91 |
| A3 | Science profits from preregistration. | 1.24 | 1.48 | 2.27 | 4.55 | 3.79 | 15.91 | 25 | 26.52 | 21.97 |
| A4 | Preregistration of studies should be obligatory. | -0.29 | 1.85 | 17.42 | 14.39 | 11.36 | 18.18 | 18.18 | 15.91 | 4.55 |
| A5 | In selection decisions people who preregister their studies should be preferred (given the same qualification). | -0.02 | 1.95 | 15.15 | 12.88 | 11.36 | 18.18 | 15.91 | 15.15 | 11.36 |
| A6 | Some researchers exploit preregistration (e.g., by cheating).* | 0.08 | 1.33 | 3.79 | 11.36 | 6.82 | 45.45 | 19.7 | 9.09 | 3.79 |
| A7 | I think that preregistration does not improve research significantly.* | -1.11 | 1.58 | 19.7 | 28.03 | 25.76 | 9.09 | 6.82 | 9.09 | 1.52 |
| A8 | Preregistration cannot prevent questionable research practices.* | 0.69 | 1.55 | 1.52 | 9.09 | 17.42 | 9.09 | 25.76 | 28.03 | 9.09 |
| A9 | Preregistration can prevent selective reporting (i.e., only reporting variables that yielded significant results). | 1.28 | 1.39 | 0.76 | 4.55 | 7.58 | 12.12 | 19.7 | 38.64 | 16.67 |
| A10 | Preregistration can prevent p-hacking (i.e., misusing data analyses to find patterns that can be presented as statistically significant). | 1.05 | 1.54 | 0.76 | 8.33 | 10.61 | 9.09 | 25.76 | 28.03 | 17.42 |
| A11 | Preregistration can prevent publication bias (i.e., only publishing positive/significant results). | 0.7 | 1.7 | 4.55 | 12.12 | 6.06 | 13.64 | 25.76 | 25 | 12.88 |
| A12 | Preregistration improves a study’s quality. | 0.65 | 1.58 | 3.79 | 9.85 | 9.85 | 12.88 | 29.55 | 25.76 | 8.33 |
| A13 | Preregistration increases the credibility of psychological research. | 1.31 | 1.47 | 1.52 | 6.06 | 6.82 | 6.06 | 23.48 | 37.12 | 18.94 |
| A14 | Preregistration is a bad initiative.* | -2.04 | 1.24 | 50 | 24.24 | 10.61 | 12.12 | 1.52 | 0.76 | 0.76 |
| A15 | The costs of preregistering a study are higher than the usefulness of preregistering it.* | -0.59 | 1.63 | 14.39 | 17.42 | 20.45 | 25 | 9.85 | 9.09 | 3.79 |
| A16 | Preregistering studies is generally unnecessary.* | -1.33 | 1.5 | 25.76 | 26.52 | 23.48 | 12.88 | 3.03 | 7.58 | 0.76 |
| A17 | Science should be open and transparent. | 2.52 | 0.81 | 0 | 0.76 | 0.76 | 0.76 | 6.06 | 26.52 | 65.15 |
| A18 | I feel that nowadays, a great variety of new structures help creating preregistrations, for example templates or repositories (i.e., uploading platforms). | 1.06 | 1.35 | 1.52 | 3.79 | 4.55 | 23.48 | 22.73 | 31.82 | 12.12 |
| A19 | Preregistration makes science more transparent. | 1.51 | 1.42 | 2.27 | 3.03 | 4.55 | 6.82 | 24.24 | 33.33 | 25.76 |
| A20 | Preregistration is very useful. | 1.07 | 1.52 | 1.52 | 7.58 | 6.06 | 15.15 | 24.24 | 28.03 | 17.42 |
| A21 | Preregistration is not useful in practice.* | -1.34 | 1.49 | 25.76 | 28.03 | 19.7 | 14.39 | 6.82 | 3.79 | 1.52 |
| A22 | Preregistration hinders exploratory research.* | -0.04 | 1.91 | 15.15 | 13.64 | 12.12 | 10.61 | 22.73 | 18.94 | 6.82 |
| A23 | Preregistration decreases scientific progress.* | -1.27 | 1.66 | 28.03 | 27.27 | 15.15 | 12.88 | 9.85 | 3.03 | 3.79 |
| A24 | A preregistration badge (i.e., a public acknowledgment that a study was preregistered provided by many journals) increases my trust in a study. | 0.52 | 1.65 | 5.3 | 12.12 | 6.82 | 15.91 | 28.03 | 24.24 | 7.58 |
| SN1 | My peers and colleagues motivate me to preregister my studies. | 0.17 | 1.57 | 5.3 | 15.15 | 6.82 | 29.55 | 21.21 | 17.42 | 4.55 |
| SN2 | I want to be part of the open science movement. | 1.41 | 1.45 | 3.03 | 2.27 | 3.03 | 13.64 | 23.48 | 29.55 | 25 |
| SN3 | My co-authors and supervisors want me to preregister my studies. | -0.06 | 1.69 | 5.3 | 21.21 | 10.61 | 29.55 | 12.88 | 11.36 | 9.09 |
| SN4 | Preregistration is highly acknowledged in psychological science. | 0.4 | 1.4 | 5.3 | 3.79 | 15.15 | 20.45 | 34.85 | 17.42 | 3.03 |
| SN5 | In today's psychological research community, it is deemed necessary to preregister. | 0.02 | 1.4 | 3.03 | 12.12 | 21.97 | 21.97 | 26.52 | 12.12 | 2.27 |
| SN6 | I feel social pressure to preregister my studies. | -0.58 | 1.59 | 13.64 | 21.97 | 13.64 | 19.7 | 22.73 | 7.58 | 0.76 |
| SN7 | I feel social pressure not to preregister my studies.* | -1.68 | 1.34 | 37.12 | 25.76 | 13.64 | 16.67 | 5.3 | 1.52 | 0 |
| SN8 | I think that many researchers preregister their studies. | -0.35 | 1.18 | 0.76 | 16.67 | 28.79 | 32.58 | 13.64 | 6.82 | 0.76 |
| PBC1 | It would be easy for me to preregister my studies. | 0.54 | 1.68 | 3.03 | 13.64 | 12.88 | 11.36 | 27.27 | 19.7 | 12.12 |
| PBC2 | I know how to create and upload a preregistration. | 1.25 | 1.9 | 3.79 | 11.36 | 8.33 | 3.79 | 12.88 | 25 | 34.85 |
| PBC3 | With the current implementation of preregistration, it is my decision if I want to preregister my studies. | 1.62 | 1.23 | 0 | 3.79 | 3.03 | 9.09 | 17.42 | 44.7 | 21.97 |
| PBC4 | Even if I wanted to preregister, there are external factors that hinder me (e.g., my co-authors or supervisors do not want to preregister).* | -0.95 | 1.64 | 20.45 | 25.76 | 12.88 | 18.94 | 15.91 | 3.03 | 3.03 |
| PBC5 | I don’t feel well informed about preregistration.* | -0.77 | 1.88 | 18.18 | 31.82 | 11.36 | 9.85 | 10.61 | 12.88 | 5.3 |
| I1 | I will preregister my studies in the near future. | 1.17 | 1.66 | 3.79 | 6.06 | 5.3 | 15.15 | 18.18 | 26.52 | 25 |
| I2 | I intend to preregister my studies in the future. | 1.21 | 1.6 | 3.79 | 5.3 | 3.03 | 15.15 | 23.48 | 25 | 24.24 |
| I3 | I want to use preregistration in the future. | 1.3 | 1.58 | 3.03 | 6.06 | 3.03 | 12.12 | 22.73 | 28.03 | 25 |
| M1 | I feel like preregistration is an investment in my future (e.g., it is helpful for my career). | 0.39 | 1.64 | 5.3 | 11.36 | 9.09 | 25 | 21.21 | 18.94 | 9.09 |
| M2 | I have the feeling that nowadays, it is harder to publish studies that were not preregistered. | -0.21 | 1.47 | 5.3 | 15.91 | 21.97 | 25 | 17.42 | 12.12 | 2.27 |
| M3 | Preregistration helps me to plan my study in more detail. | 1.29 | 1.58 | 3.79 | 5.3 | 2.27 | 12.12 | 24.24 | 28.03 | 24.24 |
| M4 | The preregistration badge (i.e., a public acknowledgment that a study was preregistered provided by many journals) would be an incentive for me to preregister my studies. | -0.16 | 1.72 | 10.61 | 18.18 | 11.36 | 17.42 | 25.76 | 12.12 | 4.55 |
| M5 | I feel morally obligated to preregister my studies. | -0.05 | 1.9 | 13.64 | 14.39 | 11.36 | 18.18 | 19.7 | 11.36 | 11.36 |
| M6 | I think planning studies in detail in the context of preregistration is fun. | 0.02 | 1.67 | 10.61 | 11.36 | 12.12 | 19.7 | 28.79 | 12.12 | 5.3 |
| M7 | Preregistration helps making my studies more transparent. | 1.48 | 1.37 | 2.27 | 3.03 | 2.27 | 9.85 | 24.24 | 35.61 | 22.73 |
| M8 | I want others to be able to comment on my planned studies. | 0.42 | 1.58 | 4.55 | 9.85 | 10.61 | 25.76 | 21.21 | 19.7 | 8.33 |
| M9 | It is necessary that preregistration limits my flexibility. | 0.27 | 1.54 | 6.06 | 6.82 | 15.15 | 28.03 | 20.45 | 17.42 | 6.06 |
| M10 | I use preregistration because it represents good scientific practice for me. | 0.92 | 1.68 | 4.55 | 8.33 | 1.52 | 23.48 | 21.97 | 18.94 | 21.21 |
| O1 | I am afraid that I could have a competitive disadvantage when I preregister my studies. | -0.73 | 1.83 | 19.7 | 23.48 | 14.39 | 16.67 | 10.61 | 9.09 | 6.06 |
| O2 | Given the present scientific context, preregistering my studies hinders my career. | -1.05 | 1.64 | 23.48 | 23.48 | 15.91 | 18.94 | 10.61 | 4.55 | 3.03 |
| O3 | I am afraid that after preregistering my studies others will find errors or deviations in/from my study plans. | -0.58 | 1.62 | 11.36 | 26.52 | 14.39 | 15.91 | 23.48 | 5.3 | 3.03 |
| O4 | I don't like that preregistration limits my flexibility. | -0.05 | 1.83 | 11.36 | 17.42 | 11.36 | 14.39 | 22.73 | 15.91 | 6.82 |
| O5 | Preregistration causes considerable time cost. | 0.98 | 1.53 | 4.55 | 3.79 | 7.58 | 10.61 | 35.61 | 22.73 | 15.15 |
| O6 | For me, there are not enough incentives to preregister my studies. | -0.08 | 1.72 | 12.12 | 11.36 | 14.39 | 20.45 | 22.73 | 13.64 | 5.3 |
| O7 | I would be afraid of scooping (i.e., someone taking my idea and publishing it before me) when preregistering my study. | -0.42 | 1.85 | 12.12 | 26.52 | 15.15 | 9.85 | 15.15 | 15.91 | 5.3 |
| O8 | I am unsure about confidentiality issues and intellectual property rights when preregistering my studies. | -0.21 | 1.76 | 9.85 | 21.21 | 10.61 | 22.73 | 17.42 | 10.61 | 7.58 |
| O9 | For my projects, preregistration is unnecessary. | -0.64 | 1.77 | 18.94 | 20.45 | 10.61 | 25 | 9.85 | 11.36 | 3.79 |
| O10 | Preregistration slows down the scientific progress of my project. | -0.09 | 1.71 | 9.09 | 13.64 | 21.21 | 16.67 | 18.18 | 15.15 | 6.06 |

Parameters of the sample that was used for the hypotheses tests, only considering participants who were recruited from the general databases (i.e., Web of Science, PubMed, PSYNDEX, and PsycInfo, *N* = 132). Items marked with * are negatively poled and were reversed for further analysis. -3 = Strongly disagree. 3 = Strongly agree.
