# Supplementary material for: Registered report: Survey on attitudes and experiences regarding preregistration in psychological research
Source: PLoS One. 2023 Mar 16;18(3):e0281086. doi: 10.1371/journal.pone.0281086 (PMC10019715; doi:10.1371/journal.pone.0281086)
Supplement: S8 Text — The results of all regression models used for the hypotheses tests are displayed in table format for easy inspection. (DOCX) [file pone.0281086.s012.docx]

Supporting information to ‘Registered Report: Survey on attitudes and experiences regarding preregistration in psychological research’:

**S16: Regression tables**

Lisa Spitzer^1^ & Stefanie Mueller^1^

^1^ Leibniz Institute for Psychology

**Table 1. Regression model of hypothesis 1.**

| **Variable** | **B** | **95% CI** | **β** | ***SE*** | ***t*** | ***p*** |
| --- | --- | --- | --- | --- | --- | --- |
| Intercept | 0.22 | 0.05, 0.38 |  | 0.08 | 2.56 | .011 |
| Attitudes | 0.65 | 0.52, 0.77 | .46 | 0.06 | 10.07 | <.001 |
| Importance | 0.3 | 0.22, 0.38 | .36 | 0.04 | 7.16 | <.001 |
| Attitudes * importance | -0.09 | -0.14, -0.05 | -.18 | 0.02 | -3.92 | > .999 |
| Subjective norm | 0.23 | 0.12, 0.35 | .15 | 0.06 | 3.87 | <.001 |
| Perceived behavioral control | 0.23 | 0.13, 0.33 | .2 | 0.05 | 4.65 | <.001 |
| Preregistration experience | 0.21 | -0.01, 0.43 | .07 | 0.11 | 1.87 | .062 |
| *F*-test | *F*(6, 282) = 137.6, *p* < .001 | | | | | |
| Adjusted *R²* | 73.99% | | | | | |

Dependent variable: Intention. For the intercept and preregistration experience, the two-sided p-value is displayed. All other predictors were tested one-sided. B = unstandardized regression weights. CI = Confidence intervals of B. *SE* = Standard errors.

**Table 2. Regression model of hypothesis 2 - attitudes**

| **Variable** | **B** | **95% CI** | **β** | ***SE*** | ***t*** | ***p*_cor_** |
| --- | --- | --- | --- | --- | --- | --- |
| Intercept | 1.05 | 0.85, 1.26 |  | 0.11 | 9.93 | <.001 |
| Research experience | -0.03 | -0.04, -0.02 | -.28 | 0.01 | -5.04 | <.001 |
| Preregistration experience | 0.5 | 0.28, 0.72 | .24 | 0.11 | 4.38 | <.001 |
| *F*-test | *F*(2, 286) = 21.18, *p* < .001 | | | | | |
| Adjusted *R²* | 12.29% | | | | | |

Dependent variable: attitude scale. All predictors were tested two-sided. B = unstandardized regression weights. CI = Confidence intervals of B. *SE* = Standard errors**.**

**Table 3. Regression model of hypothesis 2 - motivations**

| **Variable** | **B** | **95% CI** | **β** | ***SE*** | ***t*** | ***p*_cor_** |
| --- | --- | --- | --- | --- | --- | --- |
| Intercept | 0.67 | 0.47, 0.87 |  | 0.1 | 6.57 | <.001 |
| Research experience | -0.03 | -0.04, -0.02 | -.28 | 0.01 | -5.03 | <.001 |
| Preregistration experience | 0.44 | 0.23, 0.66 | .23 | 0.11 | 4.08 | <.001 |
| *F*-test | *F*(2, 286) = 19.88, *p* < .001 | | | | | |
| Adjusted *R²* | 11.59% | | | | | |

Dependent variable: motivation scale. All predictors were tested two-sided. B = unstandardized regression weights. CI = Confidence intervals of B. *SE* = Standard errors.

**Table 4. Regression model of hypothesis 2 - obstacles**

| **Variable** | **B** | **95% CI** | **β** | ***SE*** | ***t*** | ***p*_cor_** |
| --- | --- | --- | --- | --- | --- | --- |
| Intercept | 0.08 | -0.16, 0.32 |  | 0.12 | 0.66 | .51 |
| Research experience | 0.003 | -0.01, 0.02 | .03 | 0.01 | 0.49 | .623 |
| Preregistration experience | -0.81 | -1.06, -0.55 | -.35 | 0.13 | -6.26 | <.001 |
| *F*-test | *F*(2, 286) = 19.62, *p* < .001 | | | | | |
| Adjusted *R²* | 11.45% | | | | | |

Dependent variable: obstacle scale. All predictors were tested two-sided. B = unstandardized regression weights. CI = Confidence intervals of B. *SE* = Standard errors.
