# Supplementary material for: Registered report: Survey on attitudes and experiences regarding preregistration in psychological research
Source: PLoS One. 2023 Mar 16;18(3):e0281086. doi: 10.1371/journal.pone.0281086 (PMC10019715; doi:10.1371/journal.pone.0281086)
Supplement: Attachment — Submitted filename: renamed_49289.docx [file pone.0281086.s017.docx]

# COMMENTS – Manuscript PONE-D-22-30808

Title: “Registered Report: Survey on attitudes and experiences regarding preregistration in psychological research”

**Important note: This review pertains only to ‘statistical aspects’ of the study and so ‘clinical aspects’ [like medical importance, relevance of the study, ‘clinical significance and implication(s)’ of the whole study, etc.] are to be evaluated [should be assessed] separately/independently. Further please note that any ‘statistical review’ is generally done under the assumption that (such) study specific methodological [as well as execution] issues are perfectly taken care of by the investigator(s). This review is not an exception to that and so does not cover clinical aspects {however, seldom comments are made only if those issues are intimately / scientifically related & intermingle with ‘statistical aspects’ of the study}. Agreed that ‘statistical methods’ are used as just tools here, however, they are vital part of methodology [and so should be given due importance]. I look at the manuscript in/with statistical view point, other reviewer(s) look(s) at it with different angle so that in totality the review is very comprehensive. However, there should be efforts from authors side to improve (may be by taking clues from reviewer’s comments). Therefore, please do not limit the revision only (with respect) to comments made here.**

**COMMENTS:** This manuscript of Registered Report of Survey is excellent, however, I wonder to note that the protocol with same name [Registered Report Protocol (DOI: [10.1371/journal.pone.0253950](https://doi.org/10.1371/journal.pone.0253950)).] was published in July, 2021 but is mentioned directly in the ‘Methods’ section (i.e., in lines 191-92). There you said “methods and analyses reported were implemented as described in the protocol”. I find many similarities in these {this article and the protocol}. Very good that authors report them [good summary] but they are reported in supplementary file [S2 Text. Deviations from the Registered Report Protocol. This table summarizes all deviations from the preregistered procedure described in the Registered Report Protocol. For all, a description and justification are given], whereas, in my opinion, they should have been in main text.

As said in S2 “The section sampling procedure was moved down in the manuscript and divided into smaller subsections to facilitate readability of the article. In its current form, everything that was taken directly from the descriptions of the Registered Report Protocol is displayed in the beginning of the manuscript, and everything that was added (results, information about the data collection, e.g., proportion of participants recruited through each database etc.) is displayed in the second part of the manuscript” is useful. And what is said in lines 228-29 (also 534) [“Scales were recoded from “1 to 7” to “-3 to +3” for data analyses yielding a middle category which has absolute meaning”] is appreciated because that will definitely yield correct and meaningful ‘arithmetic mean’ which is useful not only for comparison but application of any statistical test(s) assumes that meaning of entity used (mean, SD, etc) has a particular meaning {which is achieved only then}.

As pointed out in ‘important note’ above “This review pertains only to ‘statistical aspects’ of the study and so ‘clinical aspects’ should be assessed separately/independently [one should carefully consider/look at the clinical implications of the study]. However, in my considered opinion, there should be no hesitation in accepting this article.
